# Production of 5-hydroxymethylfurfural from Japanese cedar (*Cryptomeria japonica*) in an ionic liquid, 1-methylimidazolium hydrogen sulfate

**DOI:** 10.1038/s41598-021-02259-2

**Published:** 2021-11-23

**Authors:** Ryoya Ito, Hisashi Miyafuji

**Affiliations:** Graduate School of Life and Environmental Sciences, Kyoto Prefectural University, Hangi-cho, Shimogamo, Sakyo-ku, Kyoto, 606-8522 Japan

**Keywords:** Bioenergy, Ionic liquids, Chemical engineering

## Abstract

Production of 5-hydroxymethylfurfural (5-HMF) from Japanese cedar (*Cryptomeria japonica*) using an ionic liquid, 1-methylimidazolium hydrogen sulfate ([MIM]HSO_4_), was investigated. 5-HMF can be produced from *C. japonica* at temperatures above 120 °C. The maximum yield of 5-HMF was about 9 wt% after 15 min of treatment at 160 °C. However, 5-HMF produced in this process tended to decompose as the treatment continued. To avoid decomposition and to provide a means of recovering 5-HMF from [MIM]HSO_4_, three reaction systems based on [MIM]HSO_4_ were investigated: biphasic [MIM]HSO_4_/organic solvent system, [MIM]HSO_4_ with vacuum distillation, and [MIM]HSO_4_ with vacuum steam distillation. The [MIM]HSO_4_ reaction system combined with vacuum steam distillation was most effective. The maximum yield of 5-HMF was 17.5 wt% after treatment for 45 min at 160 °C. The combination of [MIM]HSO_4_ treatment with vacuum steam distillation is suitable for 5-HMF production because it is a one-pot process without the need for catalysts or pretreatment.

## Introduction

In recent years, wood has been utilized to produce liquid fuels and fine chemicals^1,2^. Monosaccharides such as pentoses and hexoses are generated from cellulose and hemicelluloses in wood by hydrolysis. These monosaccharides and their derivatives can be transformed into useful building block chemicals, which are substitutes for many chemicals derived from petroleum^3^. 5-Hydroxymethylfurfural (5-HMF), which is derived from hexoses, is considered one of the most important building block chemicals^4,5^ because 2,5-dimethylfuran (DMF) and 2,5-furandicarboxylic acid (FDCA) can be produced from 5-HMF by hydrogenation and oxidation, respectively. DMF is used as a biofuel alternative for gasoline^6^. FDCA can be used in a range of applications, including the manufacture of green materials, pharmaceuticals, chemicals, and biopolymers^7,8^. FDCA is also considered to have potential as a replacement for terephthalic acid, which is used as raw material for PET production.

Production of 5-HMF from hexoses has been studied using many solvents including water, aqueous solutions of inorganic salts or acid, and organic solvents such as dimethyl sulfoxide, *N*,*N*-dimethylformamide, and sulfolane^9–13^. Frequently, fructose has been used to produce 5-HMF because 5-HMF is easily obtained from the furanose form through dehydration^14^. The formation of 5-HMF from glucose is also important because glucose exists in great quantities as a component of cellulose. However, for glucose to be converted into 5-HMF, glucose must first be isomerized to fructose before the dehydration step^15^.

Recently, formation of 5-HMF in ionic liquids has been studied. Ionic liquids are organic salts that are composed of large organic cations and inorganic or organic anions with melting points near ambient temperature. They have good thermal stability, low vapor pressure, low toxicity, and good solvation power. Ionic liquids have been suggested as part of the strategy to tackle the challenges of biorefinery or chemical processing of wood because some ionic liquids can dissolve cellulose or lignin^16–20^. In 2007, Zhao et al.^21^ processed monosaccharides in 1-ethyl-3-methylimidazolium chloride ([EMIM]Cl) with CrCl_2_ as catalyst. This reaction system led to conversions of glucose to 5-HMF with 70 mol% yield. The promising results obtained for glucose in [EMIM]Cl with CrCl_2_ have encouraged studies of lignocellulosic materials with ionic liquids^22–28^. Pine wood was converted to 5-HMF in 1-butyl-3-methylimidazolium chloride ([BMIM]Cl) with CrCl_3_·6H_2_O as catalyst in 52 mol% yield by reaction at 200 °C^29^. These studies suggest that 5-HMF can be produced from lignocellulosics without pretreatments such as delignification and hydrolysis. However, typical studies of the formation of 5-HMF from lignocellulosics in ionic liquid have involved the use of catalysts^30^. In addition, extraction of 5-HMF from ionic liquid is also important. 5-HMF is not stable in various reaction media and degrades to levulinic acid (LA) and formic acid (FA)^31,32^. Once generated, LA and FA can polymerize with 5-HMF to form humin, which is known as a by-product of saccharide-based biorefinery processes^8,12^. Our previous studies also revealed that 5-HMF produced in ionic liquids, [EMIM]Cl^33^ or 1-methylimidazolium hydrogen sulfate ([MIM]HSO_4_)^34^ as reaction medium is not stable under heating. Thus, 5-HMF should be continuously removed from the reaction medium to prevent the generation of by-products. As a fundamental research, biphasic reaction/separation coupling system was studied. In this system, conversion of glucose or fructose to 5-HMF in ionic liquids and extraction of 5-HMF produced from ionic liquids with supercritical carbon dioxide was applied^35,36^. In addition, there have been reports on a method of extracting 5-HMF from dissolved in ionic liquids using organic solvents^37^, or a method of extraction after derivatizing of 5-HMF in ionic liquids to another furan compounds^37,38^. However, extraction systems to remove 5-HMF produced from lignocellulosics in ionic liquids have received little attention. Recently, there is a study in which the reaction that produces humin from 5-HMF is not regarded as a side reaction, but is positively used to produce humin. The humin produced was revealed to show the metal ion adsorption capacity^39^.

In this study, the non-catalytic production of 5-HMF from Japanese cedar (*Cryptomeria japonica*) was studied in [MIM]HSO_4_, which is a protic ionic liquid. Protic ionic liquids lead to proton donor and acceptor sites. Furthermore, the H^+^ provided by protic ionic liquids give various applications to these ionic liquids. The production cost of protic ionic liquid is lower than that of other ionic liquids^19,40–42^. The influence of water on the production of 5-HMF was studied because wood is normally humidified at ambient atmosphere. Furthermore, extraction systems to separate 5-HMF from [MIM]HSO_4_ were also investigated.

## Results and discussion

### Formation of 5-HMF in [MIM]HSO_4_

**Figure 1:**
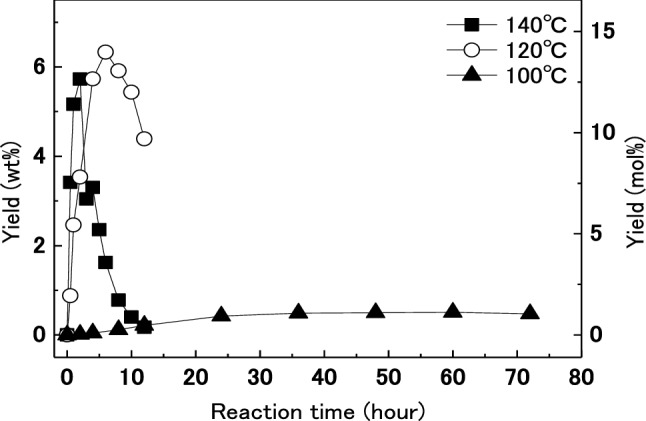
Change in yields of 5-hydroxymethylfurfural (5-HMF) with treatment time for dried wood (0.09 g) treated in [MIM]HSO_4_ (3 g) at different temperatures.

The yields of 5-HMF from dried wood in [MIM]HSO_4_ at 100, 120, and 140 °C are shown in Fig. 1. 5-HMF was produced from wood in [MIM]HSO_4_ at 120 and 140 °C, and the yield of 5-HMF reached a maximum of 6.3 wt% after treatment for 5 h at 120 °C. Given that it is known that 5-HMF degrades under acidic conditions and [MIM]HSO_4_ is an acidic ionic liquid, any 5-HMF that is produced in [MIM]HSO_4_ is likely to degrade. As the treatment temperature increased, the treatment time required to reach maximum yield became shorter and the decomposition became faster. However, lowering the treatment temperature to 100 °C showed very low production of 5-HMF.

**Figure 2:**
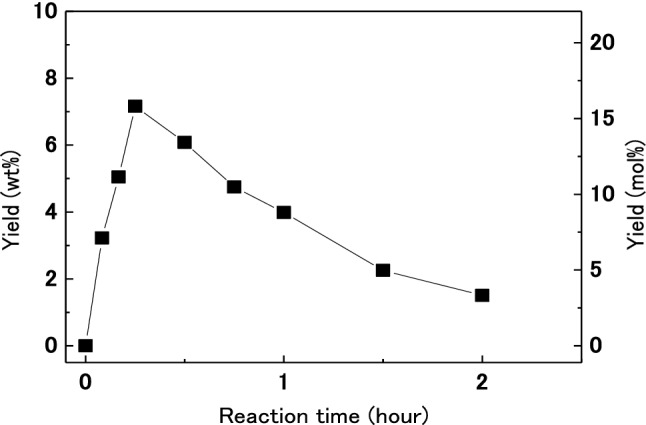
Change in yield of 5-HMF with treatment time for dried wood treated (0.09 g) in [MIM]HSO_4_ (3 g) at 160 °C.

The change in yield of 5-HMF over time for treatment of dried wood in [MIM]HSO_4_ at 160 °C is shown in Fig. 2. The yield of 5-HMF reached a maximum of 7.1 wt% after 15 min of treatment. The rate of formation of 5-HMF at 160 °C was higher than those at 100, 120 and, 140 °C as shown in Fig. 1. In addition, the maximum yield of 5-HMF at 160 °C was higher than those observed at 100, 120, and 140 °C. A similar tendency for changes in 5-HMF yield was observed in the treatment of rice straw^34^. A higher reaction temperature reduced the duration of treatment required to achieve a maximum yield. The maximum value of 5-HMF was 6.8 wt% at 160 °C around 30 min treatment.

**Figure 3:**
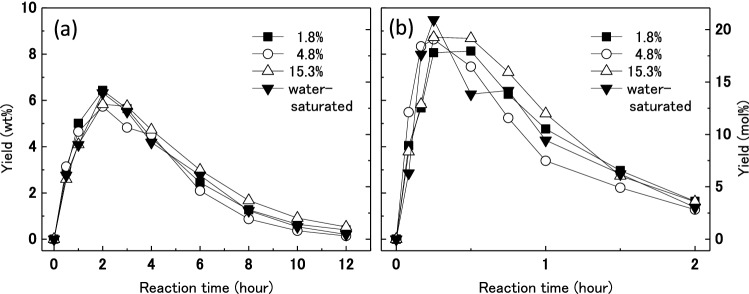
Changes of yields of 5-HMF with treatment time for humidified wood (0.09 g) treated in [MIM]HSO_4_ (3 g) at (**a**) 140 °C and (**b**) 160 °C.

It is reported that the water content in the reaction system affect the production of 5-HMF from fructose, which is different from this study in the raw material and an ionic liquid used^43^. However, to investigate the influence of water content in wood, moisture-conditioned wood was treated in [MIM]HSO_4_. Table 1 shows the moisture contents of moisture-conditioned woods after conditioning in the presence of different saturated salt solutions. These moisture-conditioned woods and water-saturated wood were treated with [MIM]HSO_4_. The changes in yield of 5-HMF over time for treatment of moisture-conditioned woods and water-saturated wood in [MIM]HSO_4_ at 140 °C and 160 °C are shown in Fig. 3. For all moisture-conditioned woods treated in [MIM]HSO_4_ at 140 °C, the yield of 5-HMF reached a maximum of about 6 wt% after 2 h (Fig. 3a). A similar trend was observed for changes in yield from the treatment of dried wood (Fig. 1). For treatment at 160 °C, the yields of 5-HMF from moisture-conditioned woods (Fig. 3b) were higher than those from dried woods (Fig. 2). The yield of 5-HMF reached a maximum of about 9 wt% after about 15 min when moisture-conditioned woods were treated in [MIM]HSO_4_ at 160 °C. Moisture-conditioned wood can be converted efficiently to 5-HMF in [MIM]HSO_4_. Given that 5-HMF is produced from glucose obtained by hydrolysis of cellulose, water is required to hydrolyze cellulose to glucose. However, 5-HMF is also produced from dried wood, so it is considered that some wood decomposition occurs, and water necessary for hydrolysis is generated from the wood during decomposition. It is speculated that water in the moisture-conditioned wood was not effectively used for the hydrolysis reaction at 140 °C, whereas the water was used for hydrolysis at 160 °C. Therefore, the temperature used to produce 5-HMF was increased to 160 °C, and moisture-conditioned wood (moisture content 4.8%) was used in subsequent experiments.

**Table 1:** Moisture contents of wood controlled by saturated inorganic salt aqueous solution.

Inorganic salt	LiCl	MgCl_2_·6H_2_O	KCl
Moisture content (%)	1.8	4.8	15.3

### Extraction of 5-HMF in [MIM]HSO_4_ by organic solvent

**Figure 4:**
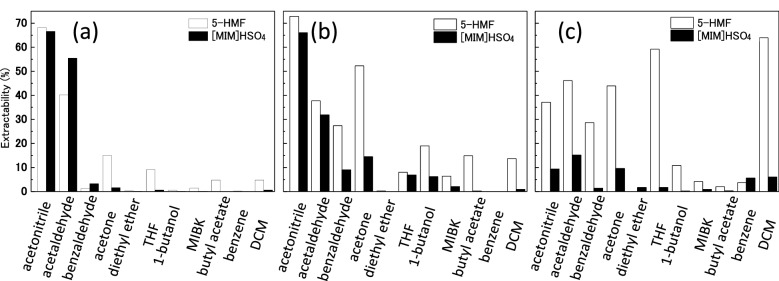
Extraction yields of 5-HMF from (**a**) reaction medium, (**b**) a mixture of [MIM]HSO_4_/water (1/1, v/v), and (**c**) a mixture of [MIM]HSO_4_/ethanol (1/1, v/v) by organic solvent. Extraction was performed on 50 μL of the reaction medium, a mixture of [MIM]HSO_4_/water (1/1, v/v), and a mixture of [MIM]HSO_4_/ethanol (1/1, v/v) with 500 μL of organic solvent. *THF* tetrahydrofuran, *MIBK* methyl isobutyl ketone, *DCM* dichloromethane.

The extraction yields of 5-HMF from the reaction medium by organic solvents are shown in Fig. 4. For reaction mixtures in [MIM]HSO_4_, 5-HMF was efficiently extracted by acetonitrile (70%) and acetaldehyde (40%) although high levels of [MIM]HSO_4_ were also extracted (70% and 55%, respectively) as shown in Fig. 4a. However, other organic solvents showed poor ability to extract 5-HMF from the reaction media. To improve the selectivity of 5-HMF extraction from the reaction medium, the reaction medium was modified by adding water or ethanol. The extraction yields of 5-HMF from the mixture of [MIM]HSO_4_ and water by organic solvents are shown in Fig. 4b. After the addition of water to the reaction mixture, acetonitrile extracted about 70% of 5-HMF, but also extracted about 65% of [MIM]HSO_4_. 5-HMF in the mixture of [MIM]HSO_4_ and water was also efficiently extracted by acetone (about 50%) with about 15% of [MIM]HSO_4_. Other organic solvents showed poor extraction of 5-HMF from the mixture of [MIM]HSO_4_ and water. The extraction yields of 5-HMF from the mixture of [MIM]HSO_4_ and ethanol by organic solvents are shown in Fig. 4c. After the addition of ethanol to the reaction mixture, 5-HMF was efficiently extracted by acetonitrile, acetaldehyde, benzaldehyde, acetone, THF, and DCM. However, in this case, [MIM]HSO_4_ was extracted with low efficiency. Of note, THF and DCM extracted 5-HMF from the mixture of [MIM]HSO_4_ and ethanol with 60% and 65% extraction yield, respectively, while [MIM]HSO_4_ extraction was very low. Among the organic solvents, THF was considered favorable because of its high extraction performance for 5-HMF and low extraction performance for [MIM]HSO_4_. In addition, THF has the advantages of low reactivity and low boiling point, which allow it to be easily separated from 5-HMF without denaturation.

### Production of 5-HMF by biphasic reaction system ([MIM]HSO_4_/organic solvent)

To continuously extract 5-HMF during the process of its formation from wood in [MIM]HSO_4_, we used a biphasic reaction system based on [MIM]HSO_4_ and an organic solvent. Table 2 shows the yields of 5-HMF extracted in the organic solvents of the biphasic reaction systems. Using benzaldehyde as the cosolvent, 5-HMF was extracted from the biphasic system in low yield at 140 and 160 °C, although 5-HMF was also present in the [MIM]HSO_4_ phase in low yield. The yield of 5-HMF extracted by benzaldehyde reached a maximum of 3.4 wt% after treatment at 160 °C for 1 h, while 5-HMF was present in the [MIM]HSO_4_ phase at 3.2 wt%. 5-HMF was considered to be unstable in benzaldehyde at 140 and 160 °C because the yield of extracted 5-HMF in benzaldehyde decreased as the reaction time extended. The extraction of 5-HMF from the biphasic system was ineffective using MIBK, butyl acetate, or toluene after treatments at 140 and 160 °C. However, in these cases, 5-HMF was still present in the [MIM]HSO_4_ phase (Table 2). In previous study, MIBK proved to be one of the most effective solvents for extraction of 5-HMF dissolved in aqueous phase^44^. In this study, however, 5-HMF is dissolved in [MIM]HSO_4_. The affinity of 5-HMF with water is thought to be different from that with [MIM]HSO_4_. Thus, the extraction by MIBK was insufficient as mentioned above. Among the organic solvents shown in Table 2, benzaldehyde was the most suitable for this reaction system. However, the use of benzaldehyde did not increase the yield of 5-HMF from wood in [MIM]HSO_4_, and removal of the solvent from the product would be particularly difficult because of its high boiling point.

**Table 2:** Yields of extracted 5-HMF from wood by biphasic ([MIM]HSO_4_/organic solvent) reaction system.

Organic solvent	Reaction temperature (°C)	Reaction time (h)	Yield (wt%)	
			Organic solvent phase	[MIM]HSO_4_ phase
Benzaldehyde	140	1	0.1	2.0
Benzaldehyde	140	2	1.2	1.3
Benzaldehyde	140	4	0.1	1.3
Benzaldehyde	160	0.5	1.6	3.7
Benzaldehyde	160	1	3.4	3.2
Benzaldehyde	160	2	0.9	0.8
MIBK	140	2	0.0	0.7
MIBK	160	0.5	0.2	1.5
Butyl acetate	140	2	0.0	4.7
Butyl acetate	160	0.5	0.2	3.0
Toluene	140	2	n.d.	3.9
Toluene	160	0.5	0.0	1.9

Extraction was performed with 6 mL of solvent on a 3 g [MIM]HSO_4_ with 0.09 g of wood (moisture content 4.8%) added.

*n.d.* not detected.

### Production of 5-HMF by vacuum distillation with [MIM]HSO_4_

To continuously recover 5-HMF from [MIM]HSO_4_ in an effort to increase 5-HMF yield, vacuum distillation was applied to the [MIM]HSO_4_ reaction system. We considered that only 5-HMF would be produced as distillate from the reaction mixture because the vapor pressure of [MIM]HSO_4_ is negligible. Table 3 shows the yields of recovered 5-HMF from wood by vacuum distillation. The yield in distillate was very low for all treatment temperatures, and most 5-HMF that was produced remained in [MIM]HSO_4_. The results showed that 5-HMF in [MIM]HSO_4_ could not be recovered by vacuum distillation, which could not effectively vaporize 5-HMF in [MIM]HSO_4_.

**Table 3:** Yields of recovered 5-HMF derived from wood (0.09 g; moisture content 4.8%) by vacuum distillation with [MIM]HSO_4_ (3 g) at 2.5 kPa.

Reaction temperature (°C)	Reaction time (min)	Yield (wt%)	
		In trap	In [MIM]HSO_4_
140	240	0.1	5.6
160	30	0.1	6.2
180	15	0.1	1.2
200	15	0.3	0.5

In another effort to improve the recovery of 5-HMF, vacuum steam distillation of the reaction system was studied. This process can effectively allow distillation of chemicals at lower temperatures. In addition, it was found that the inclusion of water in the reaction system increased the yield of 5-HMF in [MIM]HSO_4_ (see Fig. 3). Table 4 shows the yields of recovered 5-HMF from wood by using vacuum steam distillation. The yield of 5-HMF reached a maximum of 17.5 wt% after treatment at 160 °C for 45 min. This system increased the yield of 5-HMF from wood in [MIM]HSO_4_ compared with the other reaction systems used in this study. It is suggested that almost all wood was converted to 5-HMF by the treatment at 160 °C for 30 min or by the treatment at 180 °C for 20 min. The yields of 5-HMF at 200 °C were lower than those at 160 and 180 °C. However, the residues of 5-HMF at 200 °C were higher than those at 160 and 180 °C. This is because the 5-HMF produced was rapidly decomposed at 200 °C before being evaporated and converted into other compounds, which was recovered as residue. These results indicate that vacuum steam distillation at 160 and 180 °C is suitable for recovery of 5-HMF from [MIM]HSO_4_. Vacuum steam distillation is excellent not only in improving the yield of 5-HMF, but also in separating and recovering 5-HMF from [MIM]HSO_4_.

**Table 4:** Yields of recovered 5-HMF from wood (0.03 g; moisture content 4.8%) by vacuum steam distillation with [MIM]HSO_4_ (1 g) at 2.5 kPa.

Reaction temperature (°C)	Reaction time (min)	Yield (wt%)		Residue (wt%)
		In trap	In [MIM]HSO_4_	
160	15	14.9	1.3	35.7
160	30	16.4	0.1	38.1
160	45	17.5	0.1	38.4
180	10	14.1	0.6	49.6
180	20	12.2	0.1	45.8
180	30	12.2	0.0	37.0
200	5	6.6	0.5	52.1
200	10	3.8	0.5	53.9
200	15	3.9	0.0	55.7

Vacuum steam distillation is generally used for extraction and recovery of hydrophobic high-boiling-point compounds contained in solids. Given that [MIM]HSO_4_ is an ionic liquid and is a non-volatile liquid, even under reduced pressure and heating, [MIM]HSO_4_ can be considered similar to a solid. [MIM]HSO_4_ can absorb water vapor because it is hydrophilic, while 5-HMF is hydrophobic and is a high-boiling-point compound (114–116 °C at 100 Pa). These combined properties of 5-HMF and [MIM]HSO_4_ make this reaction system suitable for the extraction of 5-HMF using vacuum steam distillation.

## Materials and methods

### Sample and chemicals

**Figure 5:**
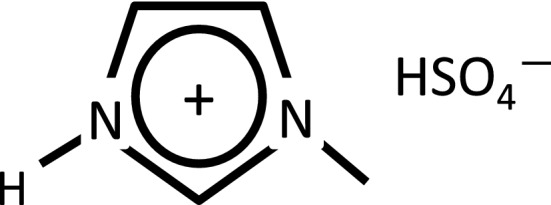
Structural formula of 1-methylimidazolium hydrogen sulfate ([MIM]HSO_4_).

1-Methylimidazolium hydrogen sulfate ([MIM]HSO_4_) as shown in Fig. 5 was purchased from Sigma-Aldrich (St. Louis, MO USA). Japanese cedar (*Cryptomeria japonica*) was collected in our university forest with permission from our university and handled in accordance with relevant guidelines and regulations. Wood flours from Japanese cedar (*Cryptomeria japonica*) were extracted with ethanol/benzene (1/2, v/v) for 24 h in a Soxhlet apparatus. The wood flours were oven-dried at 105 °C for 24 h before use. 5-Hydroxymethylfurfrural (5-HMF), LiCl, MgCl_2_·6H_2_O, KCl, ethanol, acetonitrile, acetaldehyde, benzaldehyde, acetone, diethyl ether, tetrahydrofuran (THF), 1-butanol, methyl isobutyl ketone (MIBK), butyl acetate, benzene, and dichloromethane (DCM) were purchased from Wako Pure Chemicals Industries (Osaka, Japan). In this study, an electronic balance (ATX224, Shimadzu Corporation, Kyoto, Japan; Precision: below 0.1 mg) was used for all mass measurement.

### Preparation of moisture-conditioned wood

To prepare moisture-conditioned wood, wood flours were placed in desiccators at 20 °C for 2 weeks in which the relative humidity (RH) was controlled by an aqueous saturated salt solution. The salt solutions used to control RH in the desiccators were LiCl (11.3% RH), MgCl_2_ (32.8% RH), and KCl (85.0% RH)^45^. To prepare water-saturated wood, dried wood flour was impregnated with distilled water under conditions of reduced pressure. Moisture content of moisture-conditioned wood was determined according to the equation:

1 $$ {\text{Moisture }}\;{\text{content }}\left( \% \right) \, = \, (W_{{1}} - W_{0} )/W_{0} \times { 1}00 $$

where *W*_0_ is the oven-dried mass of wood after drying at 105 °C for 24 h, and *W*_1_ is the mass of wood after moisture conditioning for 2 weeks.

### Treatment with [MIM]HSO_4_

[MIM]HSO_4_ (3 g) was heated at 100, 120, 140, or 160 °C in an oil bath with temperature controller (EO-200R, AS ONE Corporation, Osaka, Japan; Precision: ± 1 °C). Dried wood (0.09 g) or moisture-conditioned wood (0.09 g) was added to the [MIM]HSO_4_ and the reaction mixture was gently stirred. The point at which the wood flour was added to the [MIM]HSO_4_ was defined as 0 h of treatment. The treatment time was up to 72 h.

### Extraction of 5-HMF in [MIM]HSO_4_ by organic solvents

[MIM]HSO_4_ (3 g) was heated at 160 °C in an oil bath with temperature controller (EO-200R, AS ONE Corporation, Osaka, Japan; Precision: ± 1 °C). Moisture-conditioned wood (0.09 g; moisture content 4.8%) was added to the [MIM]HSO_4_ and the reaction mixture was gently stirred for 30 min. After the reaction mixture was cooled to room temperature, 50 μL of the mixture was added into 500 μL of various organic solvents and the biphasic mixtures were agitated. A 100 μL aliquot of the organic solvent phase was sampled and evaporated to dryness. The residue was then diluted with 200 μL of distilled water.

As an alternative procedure, the reaction mixture prepared as described above was mixed with water or ethanol (1/1, v/v). The mixture obtained was also extracted with organic solvents following the procedures described above.

### Production of 5-HMF in biphasic [MIM]HSO_4_/organic solvent reaction system

[MIM]HSO_4_ (3 g) was heated at 140 or 160 °C in an oil bath with temperature controller (EO-200R, AS ONE Corporation, Osaka, Japan; Precision: ± 1 °C). Moisture-conditioned wood (0.09 g; moisture content 4.8%) and 6 mL of organic solvent were added to the [MIM]HSO_4_ and the reaction mixture was gently stirred. A condenser was also connected to the reaction vessel. After treatment, 200 μL of organic phase was withdrawn and evaporated to dryness. The obtained residues were diluted with 5 mL of ethanol or 15 mL of distilled water.

### Production of 5-HMF by vacuum distillation

[MIM]HSO_4_ (3 g) was heated at 140, 160, 180, or 200 °C in an oil bath with temperature controller (EO-200R, AS ONE Corporation, Osaka, Japan; Precision: ± 1 °C). Moisture-conditioned wood (0.09 g; moisture content 4.8%) was added to the [MIM]HSO_4_ and the reaction mixture was gently stirred. The pressure in reaction system was reduced to vacuum (2.5 kPa approximately) by connection to a vacuum pump (TST-100, Sato Vac, Tokyo, Japan). The products ware recovered by cold trap cooled with ice-water. Distilled water was added to the recovered products in the cold trap to give a final volume of 20 mL.

### Production of 5-HMF by vacuum steam distillation

**Figure 6:**
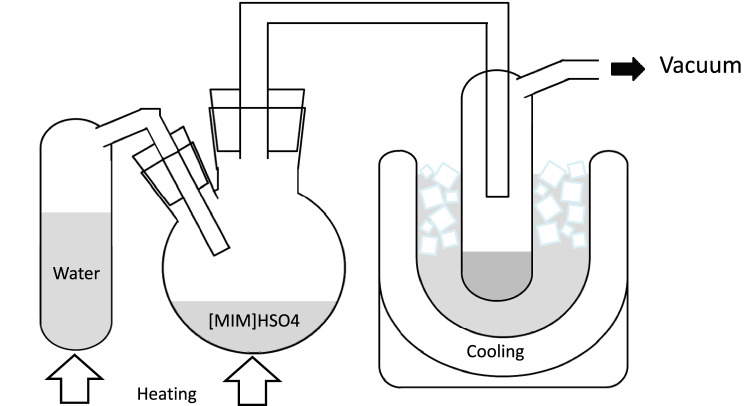
Overview of the vacuum steam distillation system used with [MIM]HSO_4_ treatment.

The configuration of the vacuum steam distillation system with [MIM]HSO_4_ is shown in Fig. 6. [MIM]HSO_4_ (1 g) was heated at 160, 180, or 200 °C in an oil bath with temperature controller (EO-200R, AS ONE Corporation, Osaka, Japan; Precision: ± 1 °C). Moisture-conditioned wood (0.03 g; moisture content 4.8%) was added to the [MIM]HSO_4_. Steam was supplied to the reaction medium, which was generated from distilled water heated at same temperature as the reaction mixture. The reduced pressure in the reaction system (2.5 kPa) was achieved by connection to a vacuum pump (TST-100, Sato Vac). The steam was recovered by a cold trap cooled with ice-water. Aqueous solution was recovered in the cold trap at 0.9 mL/min.

### Evaluation methods

5-HMF from wood in [MIM]HSO_4_ was analyzed by high-performance liquid chromatography (HPLC). The samples for analysis were prepared as follows: 40 μL of the reaction medium was mixed with 360 μL of distilled water and then filtered through a 0.45-μm filter. The filtrates were analyzed under following conditions: column, Shodex Sugar KS-801; flow rate, 1 mL/min; eluent, ultrapure water; column temperature, 80 °C; detector, refractive index detector (RID) and ultraviolet (UV) detector set at 280 nm.

Extracted and recovered 5-HMF samples and extracted [MIM]HSO_4_ were analyzed by HPLC. The samples were prepared by dilution with distilled water and were filtered through a 0.45-μm filter. The filtrates were analyzed under following conditions: column, Aminex HPX-87H (Bio-Rad); flow rate, 0.6 mL/min; eluent, 5 mM H_2_SO_4_; column temperature, 45 °C; detector, RID and UV detector set at 280 nm.

The yield of product was calculated using the following equation:

2 $${\text{Yield }}\left( {{\text{wt}}\% } \right) \, = \, ({\text{mass of produced 5-HMF}}/{\text{mass of charged sample}}) \, \times  100.$$

The molar yield of product was calculated using the following equation:

3 $$ {\text{Yield }}\left( {{\text{mol}}\% } \right) \, = \, \left( {{\text{moles}}\;{\text{ of}}\;{\text{ produced }}\;{\text{5-HMF}}/{\text{moles }}\;{\text{of}}\;{\text{ hexose }}\;{\text{unit }}\;{\text{in }}\;{\text{charged }}\;{\text{material}}} \right) \, \times { 1}00. $$

The number of moles of hexose units in Japanese cedar was determined as follows. The Japanese cedar was added to distilled water and sodium chlorite was added to the mixture to a final concentration of 0.09 M, along with a small quantity of acetic acid. The obtained reaction mixture was heated at 80 °C, and sodium chlorite with acetic acid was added to the reaction mixture every hour for 4 h. The reaction mixture was filtered and the residue, which was regarded as holocellulose, was oven-dried at 105 °C for 24 h and then weighed. The number of moles of hexose units in Japanese cedar was calculated on the basis of holocellulose and the reported composition of hexose and pentose in Japanese cedar^46^.

The extraction yield was calculated using the following equation:

4 $$ {\text{Extraction yield}}\left( \% \right) \, = \, ({\text{mass}}\;{\text{of}}\;{\text{ extracted }}\;{\text{5-HMF}}\;{\text{ or}}\;\left[ {{\text{MIM}}} \right]{\text{HSO}}_{{4}} \;{\text{by }}\;{\text{organic }}\;{\text{solvent}}/{\text{mass}}\;{\text{of}}\;{\text{ total}}\;{\text{ 5-HMF }}\;{\text{or}}\; \, \left[ {{\text{MIM}}} \right]{\text{HSO}}_{{4}} ) \, \times { 1}00 $$

In vacuum steam distillation, 10 mL of distilled water was poured into the reaction mixture to stop the reaction at a predetermined time. After stirring for 24 h at room temperature, the solution was filtered and washed with distilled water. The obtained residue was dried in an oven at 105 °C for 24 h and weighed to calculate the yield.

## Conclusions

Treatment of moisture-conditioned wood in [MIM]HSO_4_ is suitable for the production of 5-HMF without catalysts above 120 °C. However, 5-HMF is unstable in [MIM]HSO_4_ and decomposes at temperatures above 120 °C. In the biphasic reaction system with organic solvent, 5-HMF was extracted by benzaldehyde, but the yield was low and the ability to separate the solvent from 5-HMF was strongly compromised by the high boiling point of benzaldehyde. The use of vacuum distillation to separate 5-HMF from [MIM]HSO_4_ was ineffective; however, when steam was supplied to the system at 160 or 180 °C, 5-HMF was efficiently recovered as it formed in [MIM]HSO_4_. The [MIM]HSO_4_ reaction system combined with vacuum steam distillation is effective for production of 5-HMF from wood among various reaction systems studied in this paper because it is a one-pot process that requires no catalyst or pretreatment.
